# Incidental Finding of Left Ventricular False Chamber: Diagnostic and Therapeutic Implications

**DOI:** 10.1155/2018/8478475

**Published:** 2018-07-08

**Authors:** Antonello Cuccaro, Riccardo Gorla, Domenico Lumia, Mattia Barbiero, Roberto De Ponti

**Affiliations:** ^1^Department of Cardiology, Di Circolo Hospital, University of Insubria, Varese, Italy; ^2^Department of Clinical and Interventional Cardiology, IRCCS Policlinico San Donato, San Donato Milanese, Italy; ^3^Department of Radiology, Ospedale Valduce, Como, Italy; ^4^Department of Heart Surgery, Di Circolo Hospital, University of Insubria, Varese, Italy

## Abstract

We present the case of a 75-year-old man with incidental finding of a left ventricular false chamber at echocardiography. A multimodality imaging approach including also transesophageal echocardiography and cardiac magnetic resonance imaging allowed to better characterize the lesion and identify it as a pseudoaneurysm. Surgery showed an infective aetiology, which is rare, due to the finding of a large abscess in the cavity.

## 1. Introduction

Left ventricular (LV) pseudoaneurysms and true aneurysms are a rare possible consequence of myocardial infarction; other causes include cardiac surgery, infective heart diseases, and chest trauma [1]. Pseudoaneurysms form when cardiac rupture is contained by adherent pericardium or scar tissue; on the other hand, true aneurysms result from myocardial remodeling and fibrous scar formation and are composed of pericardium adherent to underlying residual fibrous scar tissue of infarcted myocardium [1]. A rare form is the annular submitral LV aneurysm that is an unusual and relatively unknown cardiac condition that occurs mostly in the black population of sub-Saharan Africa and rarely reported in the Caucasians [2].

## 2. Case Presentation

A 75-year-old man with history of percutaneous coronary intervention of proximal and distal left anterior descending (LAD) for inferolateral non-ST-elevation myocardial infarction six months earlier presented to our department for clinical follow-up. He was asymptomatic and was hospitalized due to recurrent fever four months earlier. Meningoencephalitis was suspected, but all tests were negative.

**Figure 1:**
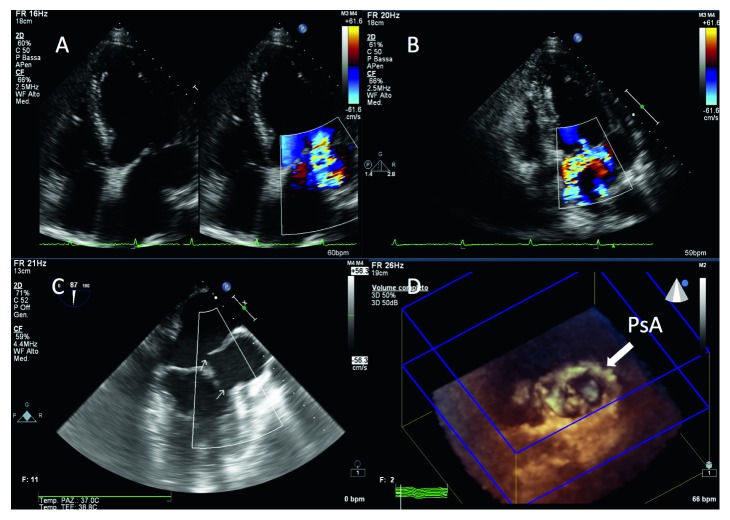
Transthoracic echocardiography showing the submitral pseudoaneurysm (A); at color Doppler, communication between the false chamber and left atrium is evident (B); transesophageal echocardiography displayed the false chamber communicating with both the left atrium and left ventricle (C, arrows); 3D transthoracic echocardiography displaying the left atrium and the false chamber (PsA) paired (D).

Transthoracic echocardiography showed a fluid-filled chamber arising from the posterolateral wall of the left ventricle, immediately below the mitral annulus (Figures 1 and 1); through the 3D echo, the left atrium and the chamber are seen paired (Figure 1). Severe mitral regurgitation and mild pericardial effusion were also evident. Laboratory parameters were unremarkable. Transesophageal echocardiography revealed a large submitral pseudoaneurysm (34 × 61 × 50 mm) communicating with left ventricle and left atrium through a single neck (Figure 1, arrows). Severe mitral regurgitation was due to partial detachment of mitral annulus.

**Figure 2:**
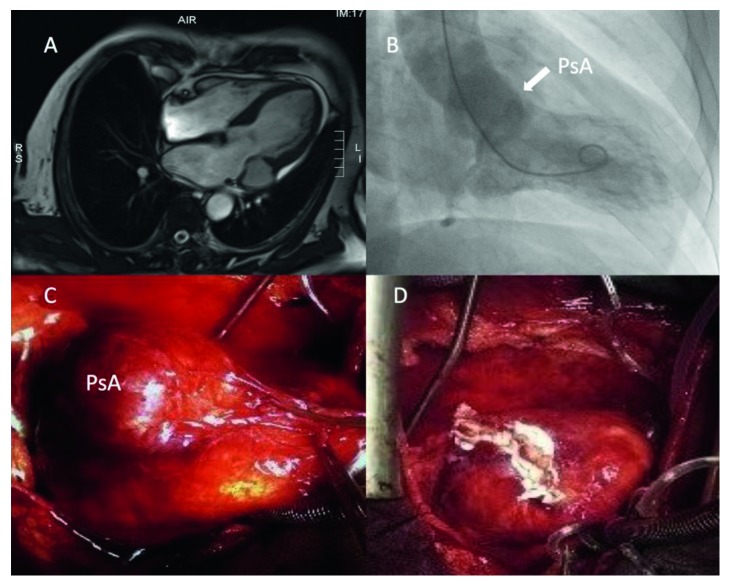
Cardiac magnetic resonance showing the pseudoaneurysm (A); the pseudoaneurysm (PsA) seen at contrast ventriculography (B, arrow); surgical view of the PsA before (C) and after resection (D).

Magnetic resonance imaging (MRI) demonstrated systolic blood flow entering the pseudoaneurysm from the left ventricle and through the cavity into the left atrium (Figure 2, video). Coronary angiography showed patency of LAD stents; contrast ventriculography confirmed the large pseudoaneurysmal cavity (Figure 2, arrow).

The patient was referred to surgery (Figures 2 and 2) which showed partial annular detachment as well as perforation of the posterior mitral leaflet at P1 scallop. Additionally, a large abscess with presence of pus was identified inside the cavity on the ventricular side of the mitral annulus, thus confirming an infective aetiology. The patient underwent surgical resection of the pseudoaneurysm and, due to the large perforation of P1, mitral valve replacement with a biological prosthesis (29 mm St. Jude). Implantation of the mitral prosthesis was performed through “U” stitches on pledgets surrounding at the level of the cavity its inferior and superior rim. Thus, complete obliteration of the abscess cavity was achieved.

Blood cultures, markers, and viral serology were negative. The postoperative period was uneventful, and the patient was discharged without complications.

## 3. Discussion

The diagnosis of LV pseudoaneurysms can be difficult because they often clinically present with nonspecific symptoms (heart failure, chest pain, and dyspnea) attributable to other causes; furthermore, in >10% of cases, patients are asymptomatic [3]. Differentiation between LV pseudoaneurysms and true aneurysms can be challenging. The salient feature that distinguishes a pseudoaneurysm from a true aneurysm is the discontinuity of the myocardium around the cavity [4]; this finding is best seen by cardiac MRI and echocardiography [1]. Furthermore, LV pseudoaneurysms are most often located in the posterior or lateral wall, and they have neck narrower than true aneurysms, which have a wide neck and usually involve the anteroapical region [4]. Contrast ventriculography can also suggest a pseudoaneurysm when a narrow neck is detected but cannot always rule it out when an aneurysmal cavity is found [4].

A correct distinction between these two clinical entities can be important also for prognostic and therapeutic reasons. True aneurysms are usually managed medically in contrast to acute postinfarction LV pseudoaneurysms (i.e., detected within 2 weeks after infarction) which require urgent surgical resection [5]. Unlike true aneurysms which have a resistant fibrotic wall, pseudoaneurysms initially consist of loose tissues and have an excessively high propensity for secondary rupture [5].

Elective surgery should be recommended also in patients with chronic LV pseudoaneurysms (i.e., discovered more than 3 moths after myocardial infarction) in the presence of symptoms such as chest pain or dyspnea, as well as in asymptomatic patients if pseudoaneurysm diameter is >3 cm or is rapidly growing at serial imaging monitoring [5].

On the other hand, asymptomatic small chronic pseudoaneurysms may be treated conservatively because their risk of rupture seems less dramatic [5].

The subvalvular types are the least common, and the aetiology of this condition is uncertain, but it seems to be associated with a congenital weakness of the posterior mitral ring [6]. In few cases, they can occur as a sequel of endocarditis. Almost 20% to 30% of the patients with native valvular endocarditis can develop abscesses or cavities, more commonly involving the aortic valve rather than the mitral valve [7]; right-sided infective endocarditis is rare in comparison with left-sided disease and they occur commonly in injection drug users [8]. This predilection is believed to be related to the following 3 factors: (1) the relatively higher pressure on the left side of the heart that produce more turbulent flow across the mitral and aortic valves predisposing them to endothelial damage; (2) the relatively higher oxygen content of the left-side circulation, which is more supportive of bacterial growth; and (3) the epidemiologically more common congenital and acquired lesions of the left heart valves [9].

Hussain et al. [10] tried to explain the phases of development of endocarditis invasion and confirm the difference between the left-sided infective endocarditis and the right-sided disease. There is an initial phase coded as “cellulitis” or “preabscess cellulitis” without pus formation or microabscess; the second phase is the “abscess” formation with macroscopic collection of pus; the third phase is the presence of “abscess cavity” with necrosis and clots, and finally, the endothelialized cavity without pus coded as “pseudoaneurysm.” Furthermore, the authors support that infective endocarditis invasion may be driven more by chamber pressure than organisms which cause the disease [10].

Patients with infective endocarditis complicated by left ventricular pseudoaneurysm have high mortality and morbidity; this condition is very rare and is more difficult to treat compared to classic infective endocarditis; therefore, prompt surgical correction is usually the treatment of choice [5]. On the other hand, in the right-sided native infective endocarditis, surgery should generally be avoided. However, it should be considered (1) in patients with right heart failure due to severe tricuspid regurgitation with poor response to diuretic therapy, (2) if organisms that are difficult to eradicate (e.g., persistent fungi) are identified or in case of bacteremia for at least 7 days despite adequate antimicrobial therapy, and (3) if tricuspid valve vegetations >20 mm that persist after recurrent pulmonary emboli are detected [11].

In our case, weakness in the posterolateral wall of LV following myocardial infarction may have contributed to the increased risk of cavity formation associated with mitral valve endocarditis.

Additionally, although our patient presented asymptomatic and the finding was incidental, an elective surgical strategy seemed the best therapeutic option due to the pseudoaneurysm size as well as the concomitant severe mitral regurgitation.

In summary, we believe three key points should be highlighted in this case. First, a multimodality imaging approach may be helpful for a correct differentiation of a submitral LV pseudoaneurysm from the rare idiopathic submitral aneurysm (mostly seen in black Africans) or postischemic true aneurysm. Second, a fistulized periannular abscess, although unfrequent, may be a possible cause of submitral LV pseudoaneurysm even in asymptomatic patients with negative inflammatory biomarkers and blood cultures. Third, a surgical strategy seems advisable in chronic asymptomatic LV pseudoaneurysms, if rapidly expanding or large, in the presence of associated endocarditis, or with concomitant indication to coronary revascularization or valve repair/replacement.

## Supplementary Materials

Supplementary Materials Video: cardiac magnetic resonance demonstrating systolic blood flow entering the pseudoaneurysm from the left ventricle and through the cavity into the left atrium.
